# Application of Response Surface Methods To Determine Conditions for Optimal Genomic Prediction

**DOI:** 10.1534/g3.117.044453

**Published:** 2017-07-18

**Authors:** Réka Howard, Alicia L. Carriquiry, William D. Beavis

**Affiliations:** * Department of Statistics, University of Nebraska, Lincoln, Nebraska 68583; † Department of Statistics, Iowa State University, Ames, Iowa 50011; ‡ Department of Agronomy, Iowa State University, Ames, Iowa 50011

**Keywords:** mixed models, machine learning, genomic prediction, epistasis

## Abstract

An epistatic genetic architecture can have a significant impact on prediction accuracies of genomic prediction (GP) methods. Machine learning methods predict traits comprised of epistatic genetic architectures more accurately than statistical methods based on additive mixed linear models. The differences between these types of GP methods suggest a diagnostic for revealing genetic architectures underlying traits of interest. In addition to genetic architecture, the performance of GP methods may be influenced by the sample size of the training population, the number of QTL, and the proportion of phenotypic variability due to genotypic variability (heritability). Possible values for these factors and the number of combinations of the factor levels that influence the performance of GP methods can be large. Thus, efficient methods for identifying combinations of factor levels that produce most accurate GPs is needed. Herein, we employ response surface methods (RSMs) to find the experimental conditions that produce the most accurate GPs. We illustrate RSM with an example of simulated doubled haploid populations and identify the combination of factors that maximize the difference between prediction accuracies of best linear unbiased prediction (BLUP) and support vector machine (SVM) GP methods. The greatest impact on the response is due to the genetic architecture of the population, heritability of the trait, and the sample size. When epistasis is responsible for all of the genotypic variance and heritability is equal to one and the sample size of the training population is large, the advantage of using the SVM method *vs.* the BLUP method is greatest. However, except for values close to the maximum, most of the response surface shows little difference between the methods. We also determined that the conditions resulting in the greatest prediction accuracy for BLUP occurred when genetic architecture consists solely of additive effects, and heritability is equal to one.

Genomic selection (GS) is an approach for improving quantitative traits through the use of genomic prediction (GP) techniques which use information provided by phenotypic values and genotypic information for individuals, lines, varieties, or hybrids in a training set to predict phenotypic values of individuals, lines, varieties, or hybrids with only genotypic information (Meuwissen *et al.* 2001). Using GP, it is possible to improve the accuracy of prediction relative to the traditional phenotypic and marker assisted selection (Lande and Thompson 1990; Bernardo and Yu 2007). GS can increase genetic gain by increasing selection intensity because many more individuals can be assigned phenotypic values than budgets will support through field assays (Heslot *et al.* 2015). There have been many statistical methods proposed for GP, and there are numerous articles evaluating these methods for sampled populations under various conditions (de los Campos *et al.* 2010; Zhao *et al.* 2012; Howard *et al.* 2014). The relative performance of the methods depends on the attributes of the training population, including sample sizes of the training and validation populations, marker density, narrow sense heritability, *etc*. Using simulation models, these attributes can be varied and their impact on prediction accuracies has been evaluated.

In a previous publication (Howard *et al.* 2014), we simulated phenotypic and genotypic information for $F_{2}$ and backcross populations for traits with heritabilities of 0.30 and 0.70. Half of the simulated data sets had only additive genetic effects, and the other half had only two-way epistatic genetic effects among 30 loci. All simulated data had phenotypic values for 1000 individuals, and genotypic values for 2000 biallelic markers. Using the simulated data, we compared the performance of 10 best linear unbiased prediction (BLUP) and four machine learning methods in terms of prediction accuracy. The measure of accuracy reported was the Pearson correlation coefficient between the simulated phenotypic value and the predicted phenotypic value. Results showed that genetic architecture had the greatest impact on estimated accuracies: machine learning methods provided higher correlations between predictions and simulated values if the genetic architectures consisted of epistatic genetic effects. BLUP methods provided no ability to predict if the genetic architecture of the trait consisted solely of epistatic effects. The results suggest an analytical diagnostic that could reveal the underlying unknown genetic architecture of a trait in experimental data. Explicitly, a comparison of estimated prediction accuracies for a given phenotype using both types of methods could reveal whether additive or epistatic effects dominate the genetic architecture of a trait. However, we did not elaborate on the conditions under which the diagnostic could be employed. It is possible that there are many conditions that could result in large differences between estimated prediction accuracies of algorithmic and linear-model methods, or it is also possible that there are very few conditions under which the differences are large. Our purpose was to systematically investigate combinations of factors that could affect the estimated prediction accuracies of both linear model-based GP and algorithmic-based GP.

GP accuracies could be influenced by sample size, number of markers, number of QTL, epistasis, and proportion of phenotypic variance attributed to variability among genotypes (heritability in the broad sense). Further, it is possible that interactions among the factors influence GP. Our objective herein is to report a strategy for identifying conditions under which the diagnostic could be employed. We determined this by constructing the response surface for accuracies of linear-model and machine learning GP methods as determined by sample size, number of markers, number of QTL, degree of epistasis, and heritability. We chose ridge regression BLUP and support vector machine (SVM) as representatives of linear-model and machine learning GP methods because Howard *et al.* (2014) demonstrated that these provided the most consistent accuracies among mixed linear-model and machine learning approaches. After constructing the response surface of estimated prediction accuracies for these five factors we employed the steepest ascent response surface method (RSM) to demonstrate experimental efficiencies that can be gained when evaluating conditions in which GP accuracies are maximized. The steepest ascent RSM is a technique that is useful for guiding the choice of factor levels to identify the optimal condition of a variable (response) dependent on several input variables. RSMs are used in many areas of science to design experiments that will identify combinations of factors that lead to an optimum response. In this manuscript, we are extending this concept to the design of simulation studies to identify combinations of genetic architecture and input factors that maximize the difference between prediction accuracies for different GP models. The intent here is to evaluate the sensitivity of the GP models to the underlying genetic architecture and design factors using RSM.

Before communicating the methods and results for the diagnostic we provide background information on RSMs. Next, we describe how the response surface for GP accuracies was simulated, and last, we illustrate how the steepest ascent RSM can be applied to efficiently determine the specific combination of factors that maximize estimated prediction accuracies of GP.

## Background

### Response surfaces and approximation methods

RSMs are used to approximate functional relationships between a response variable *y* and a set of design variables (Khuri and Mukhopadhyay 2010) which can be used to find the combination of factor levels for which the response variables are optimized. In this context, the term optimize refers to either maximize or minimize. RSMs were first introduced by Box and Wilson (1951), and are used in many experimental disciplines, including physical, biological, environmental, and chemical sciences; engineering; and economics although we are unaware of prior work on RSMs for evaluation of statistical techniques. The primary advantage of RSMs is that the number of experimental treatment combinations required to find the optimum experimental conditions can be (much) less than the total number of treatment combinations composing the entire response surface (Myers and Montgomery 1995).

**Figure 1:**
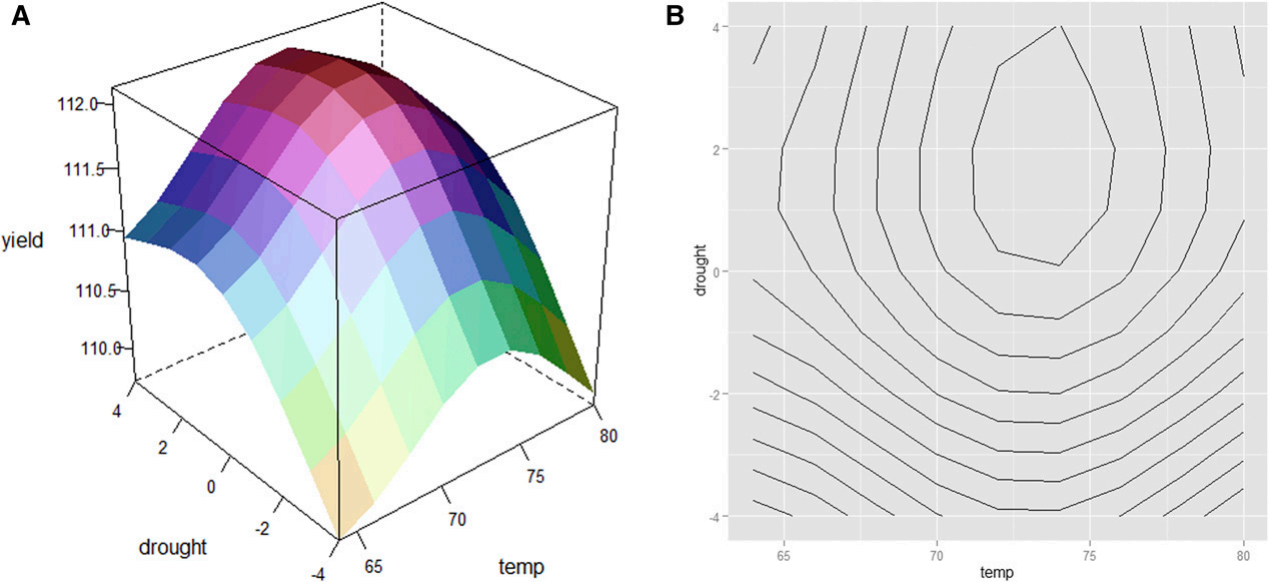
(A) The response surface of yield in relation to temperature and drought. (B) The contour plot (level curves) of the response surface of yield.

To illustrate the response surface for two variables, we simulated hypothetical yield data which are influenced by temperature and drought. Average daily temperature is simulated to be between 64 °F=18 °C and 80 °F=27 °C, and drought is between −4 and 4 standard precipitation index (SPI). Negative values for the drought index indicate conditions that are dryer than normal. The model we used to simulate yield $\;=110+\text{cos}{\left(0.25\;\text{drought}\right)}^{2}+\sin{\left(0.15\;\text{temperature}\right)}^{2}+0.0024375\;\text{drought}\times \text{temperature}.$ Figure 1A shows the response surface, and Figure 1B shows the contour plot of the simulated yield. The simulation was performed in R (R Development Core Team 2008, http://www.r-project.org) and the code can be found in Supplemental Material, File S1.

For these simulated data it is clear that the yield is maximized when temperature is between 73 °F=22.78 °C and 74 °F=23.33 °C and drought is ∼2 SPI. In most situations, the true response surface is unknown, and is influenced by more than two design variables where visualization of the data are difficult.

A model for the relationship between response *y* and the *p* design variables, $z_{1},$ $z_{2},$…, $z_{p},$ can be written in the form

$$y=f\left(z_{1},z_{2},\ldots ,z_{p}\right)+\epsilon ,$$ (1)

where *f* is the true, unknown response function and *ϵ* is the error term (Myers and Montgomery 1995). The error term is often assumed to have a normal distribution with mean 0 and variance $\sigma^{2},$ although other distributions can be modeled. If we assume that *ϵ* has a distribution that has mean 0, then the expected value of the response in terms of the natural variables can be written as

$$E\left(y\right)=E\left[f\left(z_{1},z_{2},\ldots ,z_{p}\right)+\epsilon \right]$$ (2)

$$=E\left[f\left(z_{1},z_{2},\ldots ,z_{p}\right)\right]+E\left[\epsilon \right]$$ (3)

$$=f\left(z_{1},z_{2},\ldots ,z_{p}\right).$$ (4)

The design variables $z_{1},z_{2},\ldots ,z_{p}$ are commonly referred to as natural variables because they have the natural units of the measurements. In an RSM the natural variables are transformed into coded variables: $x_{1},x_{2},\ldots ,x_{p}.$ All of the coded variables have mean 0 and the same variances. It is convenient to code the low level of the factor variables as −1, and the high level as 1.

Since the true response function is unknown, we have to approximate *f*. Under standard smoothness assumptions, a low-order polynomial function provides a good local approximation to the true *f*. For example, a first-order main effects model can be written as

$$E\left(y\right)=\beta_{0}+\beta_{1}x_{1}+\beta_{2}x_{2}+\ldots +\beta_{p}x_{p},$$ (5)

where $x_{1},x_{2},\ldots ,x_{p}$ are coded variables, .. is the unknown intercept, and $\beta_{1},\beta_{2},\ldots ,\beta_{p}$ are the unknown regression coefficients. Equation 5 is called a main effects linear model because it only contains the linear effects of the *p* factors on the response with no interaction terms. When the model in Equation 5 includes interactions, we call it the first-order model with interaction, and write it as

$$E\left(y\right)=\beta_{0}+\beta_{1}x_{1}+\beta_{2}x_{2}+\ldots +\beta_{p}x_{p}+\beta_{12}x_{1}x_{2}+\ldots ,$$ (6)

In situations such as those illustrated in Figure 1, a second-order model can be also used to model the unknown response function *f*. A second-order polynomial provides a good local approximation to almost any surface because it can have different functional forms and it is easy to estimate its parameters. In general, the second-order model can be written as

$$E\left(y\right)=\beta_{0}+\sum_{j=1}^{p}\beta_{j}x_{j}+\sum_{j=1}^{p}\beta_{jj}x_{j}^{2}+\sum \sum_{i<j\leq p}\beta_{ij}x_{i}x_{j}+\ldots$$ (7)

Let *β* denote the vector of unknown regression coefficients with dimension depending on the model. With an interaction term or a second-order model we can introduce curvature into the estimated surface. The model equation can be written in a concise matrix notation as

y=Xβ+ϵ, (8)

where **y** is an (n×1) vector of observations, **X** is an (n×p) dimensional matrix of the levels of the coded explanatory variables, *β* is a (p×1) vector of the coefficients, and *ϵ* is an (n×1) vector of the random error terms. An ordinary least-squared estimator of the model coefficients can be written as

$$\hat{b}={\left(X^{\prime }X\right)}^{-1}X^{\prime }y$$ (9)

with the variance–covariance matrix of $\hat{b}$ having the form

$$\mathrm{Var}\left(\hat{b}\right)=\sigma^{2}{\left(X^{\prime }X\right)}^{-1},$$ (10)

where $\sigma^{2}\text{I}$ is the variance-covariance matrix of ϵ. To estimate regression coefficients, *β* in the response function requires data from experiments designed to meet the objective (Myers and Montgomery 1995). If the objective is to approximate the response surface, a frequently used treatment design is the factorial. For example, temperature and the degree of drought could be two factors affecting yield, but the number of factors could be more than two and the possible values per factor can be qualitative, quantitative, and numerous. For the example illustrated in Figure 1, a reasonable full factorial model would consist of 4×5=20 factor combinations. To observe a response at each factor combination when there are two levels for the *p* factors, $2^{p}$ unreplicated treatment combinations are required and the design is called the $2^{p}$ factorial design. When *p* is large and the range of possible values of each factor is also large, finding the combination of the *p* factors needed to approximate the response surface increases exponentially. For example, three factors with two levels each requires $2^{3}=8$ treatment combinations; whereas if the number of factors is five, the number of factor combinations is $2^{5}=32.$ At least some of the treatment combinations would need to be replicated if we want an estimate of the variance of residuals, $\sigma_{\epsilon }^{2}.$

### Efficient designs to find the optimal response

Often the research objective is to identify conditions where the response is optimal, not to characterize the entire response surface. For such an objective, an experimental strategy needs only to determine the combinations of factors that optimize the response. For example, it is possible to find the maximum yield due to temperature and drought with as few as six, instead of 20, treatment combinations. Conceptually, these strategies are based on the sequential evaluation of subsets of the full factorial design. The key is to design the subsets of four-factor combinations in a manner that will maximize the information about the direction and distance to the optimum from data collected on the responses.

Choice of initial subsets of factor combinations are based on recognition that in a $2^{p}$ factorial design, *p* degrees of freedom from a total $2^{p}-1$ are used to estimate the main effects while the remaining degrees of freedom are used to estimate interactions among the factors. In an initial subset of factor combinations, it is reasonable to assume that the second-order and nonlinear aspects of the response surface are not of initial interest and use a fractional factorial design. For example, consider the half of the full factorial design also known as the $2^{p-1}$ design for a response that is influenced by three factors, *e.g.*, temperature, drought, and fertilizer, denoted A,B, and *C*. The half-factorial design in this case requires $2^{3-1}=2^{2}=4$ treatment combinations. Even though this first exploratory experiment is less expensive than the complete factorial experiment, the fractional-factorial design will involve aliasing of factors. In other words, some effects are confounded and cannot be estimated independently. In this case of the $2^{3-1}$ design, the main effects are confounded with the two-factor interactions; a subsequent experiment is needed to disentangle the confounded effects. Explicitly, consider all of the treatment combinations a,b,c,abc,ab,ac,bc, and (1) in a $2^{3}$ design (Table 1). The factorial effects are I,A,B,C,AB,AC,BC,ABC, where *I* is the identity column used to estimate the average response. The − symbol stands for a low level (level 1) of a factor, and + stands for a high level (level 2) of a factor.

**Table 1:** Combinations and factorial effects for a $2^{3}$ design

Treatment Combination	Factorial Effect							
	*I*	*A*	*B*	*C*	*AB*	*AC*	*BC*	*ABC*
*a*	+	+	−	−	−	−	+	+
*b*	+	−	+	−	−	+	−	+
*c*	+	−	−	+	+	−	−	+
*abc*	+	+	+	+	+	+	+	+
*ab*	+	+	+	−	+	−	−	−
*ac*	+	+	−	+	−	+	−	−
*bc*	+	−	+	+	−	−	+	−
(1)	+	−	−	−	+	+	+	−

Factor levels are denoted + and −. Taking only the treatment combinations where the ABC factorial effect is + or − will provide a half, *i.e.*, $2^{3-1}$ fractional-factorial design.

The identity column, *I*, is always +, and we can write

I=ABC. (11)

Equation 11 is called the defining relation for the design, and represents the relationship between the identity and a factorial effect, which determines the aliasing pattern. Multiplying both sides of (11) by *C* results in

$$C\times I=C\times ABC=ABC^{2}.$$ (12)

However, the square of any column (factorial effect) is the identity *I*, so we get

C=AB. (13)

Using the defining relation, the factorial effect of factor *C* for every treatment combination can be determined. To create a $2^{3-1}$ fractional-factorial design we can consider the treatment combinations where the ABC factorial effect has a + sign. Note that these are a,b,c, and abc treatment combinations listed in the top half of Table 1. Also, we can consider the four treatment combinations where the corresponding ABC factorial effect has a − sign. We call this the complementary fraction. These treatment combinations are the ab,ac,bc, and (1), thus illustrating the confounding interaction effects with the main effects. It does not matter which fraction we take because both belong to the same family of treatment designs.

After designing and conducting the fractional-factorial experiment for the four treatment combinations, at least four responses will provide information about the next set of treatments that should be conducted to increase the value of the response. One algorithm to do so is *steepest ascent* (or *steepest descent*). Steepest ascent is a sequential approach for finding the maximum response, where we search for a region of the factor space where the response is improved. The method of steepest ascent has three main steps (Myers and Montgomery 1995):

1. Design of factor combinations with replicates.
2. Model building.
3. Sequential experimentation.

Since the method of steepest ascent is a sequential procedure, the three steps are typically repeated until the optimum response is obtained. That is, steepest ascent can involve several experiments consisting of subsets of factor combinations that lead to the maximum response. The subsets of factor combinations in each experiment depend on the estimates of the regression coefficients of the model from prior experiments. For illustration, consider a first-order regression model

$$\hat{y}=b_{0}+b_{1}x_{1}+b_{2}x_{2}+\ldots +b_{p}x_{p}.$$ (14)

Changing values of $x_{i}\left(i=1,2,\ldots ,p\right)$ relative to the other factors depends on the estimated regression coefficient $b_{i}.$ The magnitude of $b_{i}$ provides the rate or number of steps relative to the $x_{i}$ coordinate, and the sign of $b_{i}$ tells us the direction for the next set of factor levels. If, for example, the magnitude of $b_{1}$ is twice as much as the magnitude of $b_{2},$ then $x_{1}$ will change twice as fast as $x_{2}$ for the same change in factor levels.

Simple first-order models are typically used in the initial experiments. Unless the surface is complex or the initial fractional factorial uses levels that are far from the optimal region, only a few steps will be needed to move quickly into “the neighborhood” of the optimum response. As factor combinations approach the optimum, second-order models that include interaction terms and curvature are employed to determine more accurate approximations of the underlying surface.

## Methods

### Simulated response surface

Doubled haploid (DH) populations were simulated using R (R Development Core Team 2008, http://www.r-project.org). The R code for performing the simulations, and the predictions, can be found in File S2. Since our objective is to demonstrate application of steepest ascent to determine the maximum response, we first simulated the entire response surface using a list of five factors (Table 2) with operability regions specified by factor levels (Table 3). For the number of segregating progeny, number of markers, number of QTL, and level of heritability three levels were evaluated, and for epistasis five levels were evaluated. Epistasis at level 0 means that all of the genetic variance is additive, 0.5 epistasis means that half of the genetic variance is additive and the other half is epistatic. Thus, the response surface is characterized by the 3×3×3×5×3=405 factor combinations (Table 3).

**Table 2:** Possible values for *n*, *m*, *QTL*, *epi*, and *h*

Factor	Minimum	Maximum	Other Constraints
*n*	0	∞	*n* is an integer
*m*	0	∞	*m* is an integer
*QTL*	0	∞	qtl is an integer, qtl ≤ *m*
*epi*	0	1	
*h*	0	1	

*n*, number of segregating progeny; *m*, number of markers; *QTL*, number of QTL; *epi*, proportion of genetic variance due to epistasis; *h*, heritability in the broad sense.

**Table 3:** Specification of the factors including *n*, *m*, *QTL*, *epi*, and *h*

Factor	Level 1	Level 2	Level 3	Level 4	Level 5
*n*	200	1000	2000		
*m*	100	400	1000		
*QTL*	10	50	100		
*epi*	0	0.2	0.5	0.8	1
*h*	0.2	0.5	0.8		

*n*, number of segregating progeny; *m*, number of markers; *QTL*, number of QTL; *epi*, proportion of genetic variance due to epistasis; *h*, heritability.

The simulated genomes of the DHs had 10 chromosomes, each having the same length. The markers were distributed throughout the genome in such a way that each chromosome had the same number of markers equally spaced along the length of each chromosome. There were no missing genotypic values and no missing phenotypic values. The recombination rate was simulated as a function of the number of the marker loci within a linkage group. The phenotypic values are simulated based on the model described as

$$\text{Pheno}=\mu +X_{a}a+X_{e}\mathrm{epi}+\epsilon ,$$ (15)

where Pheno is a vector of length *n* (*n* is the number of segregating progeny), $X_{a}$ is an (n×q)-dimensional additive incidence matrix where *q* is the number of QTL, **a** is a *q*-long vector of additive effects, $X_{e}$ is the (n×2q)-dimensional epistatic incidence matrix, **epi** is a 2*q*-long vector of epistatic effects, and *ϵ* is an *n*-long vector of random errors. *ϵ* has a normal distribution with mean of 0 and variance determined by h (proportion of phenotypic variance due to genetic variance) and the genetic variance. **a** and **epi** are simulated in such a way that a=aI and epi=epiI where *a* and epi are constants, and **I** is the identity vector of length specified by the model.

The genetic variance is defined as

$$V_{G}=a^{2}V_{a}+epi^{2}V_{epi}+2aepi\mathrm{Cov}\left(X_{a},X_{epi}\right),$$ (16)

where $V_{a}$ is the additive genetic variance and $V_{epi}$ is the epistatic genetic variance. The genotypic values were coded according to Cockerham’s model (Kao and Zeng 2002), and epistatic interactions were simulated between pairs of neighboring QTL. Thus, we only considered two-way gene interactions among QTL. The proportion of genetic variance explained by the additive and epistatic parts can be specified by the user of the simulation software, and is determined by using Equation 16.

To determine the response, *i.e.*, accuracy of prediction at all 405 factor and factor level combinations, phenotypes were predicted using ridge regression BLUP and SVM methods. BLUP is a parametric statistical procedure for prediction consisting of a random effect term for the marker genotypes (Henderson *et al.* 1959; Henderson 1963; Bernardo 1994; Howard *et al.* 2014). SVM is a nonparametric machine learning technique that can model the relationship between the marker values and the phenotypes using a linear or a nonlinear mapping function (Vapnik 1995; Hastie *et al.* 2009; Howard *et al.* 2014). Specifically, we used RRBLUP (Endelman 2011) and SVM (Karatzoglou *et al.* 2004) implemented in R (R Development Core Team 2008, http://www.r-project.org). Predictive accuracies were estimated using cross-validation, where the data were divided into training and testing sets. Let $ph_{i}$ denote the true phenotypic values at factor combination *i*, and let $\hat{ph_{i}}$ denote the estimated phenotypic values at factor combination *i*. Accuracy of prediction $r\left(ph,\hat{ph}\right)$ is defined as the correlation between the true phenotypic values and the predicted phenotypic values (Howard *et al.* 2014). Prediction accuracies were estimated at each treatment combination with 500 replications consisting of 20 different genotypic–phenotypic data sets, and within each data set we divided the marker and phenotypic data into 25 different training–testing subsets. The training–testing data sets were created in a such way that a random 20% of the individuals belong to the testing set, and the remaining 80% belong to the training set. We looked at the difference in prediction accuracy for SVM and BLUP techniques, and the prediction accuracy for BLUP.

### Data availability

The simulation code that produces Figure 1 can be found in File S1, and the code for simulations and predictions can be found in File S2.

## Results

### Response surfaces

**Figure 2:**
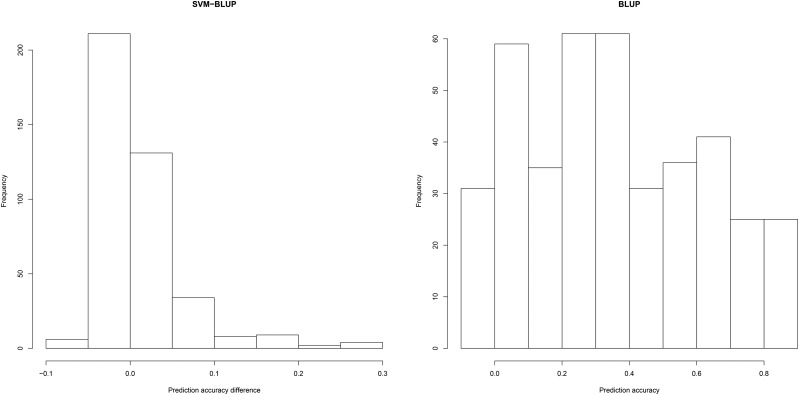
Histogram of differences of prediction accuracies between SVM and BLUP shown in the left panel. Histogram of prediction accuracies for BLUP shown in the right panel.

Three response surfaces were generated: one based on prediction accuracies of BLUP, $r_{BLUP};$ a second based on prediction accuracies of SVM, $r_{SVM};$ and the third based on the differences of the prediction accuracies, $\left[r_{SVM}-r_{BLUP}\right].$ The left panel of Figure 2 is a histogram of the differences in the prediction accuracies, $\left[r_{SVM}-r_{BLUP}\right],$ and the right panel is a histogram for $r_{BLUP}.$ The histograms are based on average prediction accuracies from 500 replicates for all 405 factor combinations (Table S1).

Most factor combinations produce similar prediction accuracies (left panel of Figure 2), although there are treatment combinations where SVM is more accurate than BLUP. The maximum difference between SVM and BLUP is 0.3, and this occurs when the number of segregating progeny is 2000 (which is the maximum we considered), the number of markers is 100 (which is the minimum we considered), h is one, and the proportion of epistatic variance relative to the total genotypic variance is one. For only 1.5% of the treatment combinations, the difference between the prediction accuracies for SVM and BLUP is >0.20, and for all of these cases epistasis and h are at their maximum limits, and the numbers of segregating progenies are 2000. Note that $r_{BLUP}$ has a wide range of values depending on the treatment combinations (Figure 2). The maximum $r_{BLUP}$ is 0.80 when the number of segregating progeny is 2000, h is at its maximum limit, and the proportion of epistatic variability accounting for the genetic variance is zero. For 6% of the treatment combinations, the prediction accuracy is >0.80 and in all those cases the number of segregating progeny is 2000, h is one, and the proportion of epistatic variability accounting for the genetic variance is zero. There was no pattern detected for the number of markers and the number of QTL (Table S1).

### Steepest ascent to determine factor combinations for optimal responses

In the steepest ascent RSM, our goal is to find the combination of factor levels associated with the optimal response *y* without evaluating every possible factor combination of the response surface. A full factorial design using five factors with two factor levels would imply $2^{5}=32$ experimental treatment combinations. Several different starting points for the factors could influence the results. Initial levels for each factor had no impact on the final estimate of the maximum response (data not shown). However, initial values did impact the number of subsets of factor combinations that were needed to reach the maximum. For the sake of brevity, we provide results of the dynamic decision process for only one set of initial values that were located furthest from the optimum (Table 4). Let $y_{1}$ be the difference between the accuracy of prediction using the BLUP and the SVM method. Let $y_{2}$ be the prediction accuracy for the BLUP method. Since the calculations for SVM are comparable to the calculations for BLUP, we only included $y_{1}$ and $y_{2}$ as responses. The response depends on the set of design variables $x_{ind},$ $x_{m},$ $x_{QTL},$ $x_{epi},$ and $x_{h};$ where $x_{ind}$ is the number of individuals in the simulated DH population, $x_{m}$ is the number of markers, $x_{QTL}$ is the number of QTL, $x_{epi}$ is the proportion of genetic variability due to epistasis, and $x_{h}$ is the proportion of phenotypic variance due to genetic variance. A model can be written as

$$y=f\left(x_{ind},x_{m},x_{QTL},x_{epi},x_{h}\right)+\epsilon ,$$ (17)

where *y* is the response; *f* is the unknown, possibly complex response function, which depends on the design variables $x_{ind},$ $x_{m},$ $x_{QTL},$ $x_{epi},$ and $x_{h};$ and ϵ∼ iid $N\left(0,\sigma_{e}^{2}\right).$ The expected value of the response function can be written as

$$E\left(y\right)=E\left[f\left(x_{ind},x_{m},x_{QTL},x_{epi},x_{h}\right)+\epsilon \right]$$ (18)

$$=f\left(x_{ind},x_{m},x_{QTL},x_{epi},x_{h}\right).$$ (19)

Initially, use a first-order polynomial to approximate the response function, *f*, so that

$$E\left(y\right)\;=\;\beta_{0}+\beta_{1}x_{ind}+\beta_{2}x_{m}+\beta_{3}x_{QTL}+\beta_{4}x_{epi}+\beta_{5}x_{h},$$ (20)

where $\beta_{0}$ is the intercept, $\beta_{1}$ is the regression coefficient associated with the number of individuals, $\beta_{2}$ is the regression coefficient associated with the number of markers, $\beta_{3}$ is the regression coefficient associated with the number of QTL, $\beta_{4}$ is the regression coefficient associated with the proportion of genetic variation due to epistasis, and $\beta_{5}$ is the regression coefficient associated with the proportion of phenotypic variability due to genotypic variability.

**Table 4:** Starting values for the factors *ind*, *m*, *QTL*, *epi*, and *h* in terms of natural units and coded units

Factor	Natural Units		Coded Units	
	Low Level	High Level	Low Level	High Level
*ind*	200	1000	−1	1
*m*	100	400	−1	1
*QTL*	10	100	−1	1
*epi*	0.2	0.5	−1	1
*h*	0.2	0.5	−1	1

*ind*, number of progeny; *m*, number of markers; *QTL*, number of QTL; *epi*, proportion of genetic variability explained by the epistatic variability *vs.* the additive variability; *h*, proportion of phenotypic variability explained by the genetic variability.

Average responses $r_{SVM},$ $r_{BLUP},$ and their difference from 500 replicates of the half-fractional factorial of 16 factor combinations (Table 5) were used to determine subsequent subsets of factor combinations that should be close to the optimum response.

**Table 5:** Mean accuracy of BLUP, mean accuracy of SVM, and the difference between the mean accuracy of SVM and mean accuracy of BLUP for 16 combinations of factors

Treatment Combination	BLUP Accuracy	SVM Accuracy	Response
1000 *ind*, 400 *m*, 100 *QTL*, 0.2 *epi*, 0.5 *h*	0.59	0.58	−0.01
200 *ind*, 100 *m*, 100 *QTL*, 0.2 *epi*, 0.5 *h*	0.53	0.54	0.01
200 *ind*, 400 *m*, 10 *QTL*, 0.2 *epi*, 0.5 *h*	0.39	0.36	−0.03
1000 *ind*, 100 *m*, 10 *QTL*, 0.2 *epi*, 0.5 *h*	0.59	0.58	−0.01
200 *ind*, 400 *m*, 100 *QTL*, 0.5 *epi*, 0.5 *h*	0.40	0.39	−0.01
1000 *ind*, 100 *m*, 100 *QTL*, 0.5 *epi*, 0.5 *h*	0.45	0.44	−0.01
1000 *ind*, 400 *m*, 10 *QTL*, 0.5 *epi*, 0.5 *h*	0.41	0.41	0.00
200 *ind*, 100 *m*, 10 *QTL*, 0.5 *epi*, 0.5 *h*	0.33	0.31	−0.02
200 *ind*, 400 *m*, 100 *QTL*, 0.2 *epi*, 0.2 *h*	0.31	0.29	−0.02
1000 *ind*, 100 *m*, 100 *QTL*, 0.2 *epi*, 0.2 *h*	0.36	0.34	−0.02
1000 *ind*, 400 *m*, 10 *QTL*, 0.2 *epi*, 0.2 *h*	0.29	0.27	−0.02
200 *ind*, 100 *m*, 10 *QTL*, 0.2 *epi*, 0.2 *h*	0.21	0.20	−0.01
1000 *ind*, 400 *m*, 100 *QTL*, 0.5 *epi*, 0.2 *h*	0.27	0.24	−0.03
200 *ind*, 100 *m*, 100 *QTL*, 0.5 *epi*, 0.2 *h*	0.18	0.15	−0.03
200 *ind*, 400 *m*, 10 *QTL*, 0.5 *epi*, 0.2 *h*	0.06	0.05	−0.01
1000 *ind*, 100 *m*, 10 *QTL*, 0.5 *epi*, 0.2 *h*	0.23	0.22	−0.01

The mean accuracy difference between the SVM and BLUP, and the estimates of the regression coefficients in terms of the coded units for the response $r_{SVM}-r_{BLUP}$ for the half factorial were

$${\hat{y}}_{1}=-0.130+0.019ind-0.004m-0.003QTL+0.043epi+0.020h.$$ (21)

Based on the estimated coefficients, increasing the number of progeny, the proportion of total genetic variance due to epistasis, and the heritability, and decreasing the number of markers and QTL should improve the response. The coefficients with the smallest magnitudes include number of markers and QTL. Thus, these are not as influential as the other factors in terms of the next set of factor combinations which will improve the response ($r_{SVM}-r_{BLUP}$).

To determine the next set of factor combinations, we define a basis and calculate the step sizes (increments) for each factor. Table 6 shows the low and high levels (level 1 and level 2) of the factors, the average of the two levels of the factors (which is the *base* in later calculations), and the distance between the average and either of the initial values (which is used for calculating the coordinates of the response surface which need to be evaluated next).

**Table 6:** Levels of factors, average of the levels of the factors, and half of the difference between the levels of factors

Factor	Level 1	Level 2	(Level 1+Level 2)/2	[(Level 1+Level 2)/2]−Level 1
*ind*	200	1000	600	400
*m*	100	400	250	150
*QTL*	10	100	55	45
*epi*	0.2	0.5	0.35	0.15
*h*	0.2	0.5	0.35	0.15

For establishing which treatment combinations need to be evaluated next to find the maximum response, the step size of the input variables needs to be determined. First, an input variable needs to be chosen (called the basis) which will also influence the step size of the other variables. It is beneficial to choose a variable for which the most information is available, but it is also a common practice to choose the basis with the largest absolute value of the estimated regression coefficient.

Because in our example the fitted model has the largest absolute estimated coefficient for epistasis, epistasis is the basis. We choose the step size for epistasis to be 0.25 because previous research indicated that the maximum response ($r_{SVM}-r_{BLUP}$) can be found when epistasis is high. The step size will only determine the number of additional experiments we have to run to reach the optimum. If we were to specify a smaller step size for epistasis, we would move more slowly on the surface by evaluating more treatment combinations. However, when the surface is not smooth and is complex, it might be beneficial to choose a step size for the first input variable that is small. The choice of the step size of the first input variable is determined by the researcher, and it influences the step size of the other input variables.

The step size of 0.25 for epistasis in natural units corresponds to 0.25/0.15=1.6667 in coded units (the value of 0.15 comes from Table 6). The step sizes for the other input variables in coded units are

$$\left(\frac{{\hat{\beta }}_{factori}}{{\hat{\beta }}_{epi}}\right)\hat{{\text{step size}}_{epi}},$$ (22)

explicitly:

$$\left(\frac{0.019}{0.043}\right)1.667=0.74$$ (23)

for individuals,

$$\left(\frac{-0.004}{0.043}\right)1.667=-0.16$$ (24)

for markers,

$$\left(\frac{-0.003}{0.043}\right)1.667=-0.12$$ (25)

for QTL, and

$$\left(\frac{0.020}{0.043}\right)1.667=0.78$$ (26)

for h.

To determine the coordinates of the response surface (*i.e.*, experimental conditions) that need to be evaluated next, we need to convert the step sizes in coded units into natural units. The step size in natural units can be calculated as the product of [(level 1+level 2)/2]−level 1 and the corresponding step size in coded units (Table 7). For example, for the number of individuals the step size in natural units is calculated as 400(0.74). The base in natural units for the input variables corresponds to a base of zero in coded units. Δ, 2Δ, 3Δ, … indicate the direction and magnitude of change for each factor which can be used for the next set of experimental conditions; and base+Δ, base+2Δ, base+3Δ, … specify the coordinates of the response surface that need to be evaluated next (Table 8).

**Table 7:** Base, step size in natural units, and the coordinates of the steepest ascent for the number of individuals, number of markers, number of QTL, proportion of epistasis, and the degree of heritability to determine the second set of factor combinations for response $r_{SVM}-r_{BLUP}$

	Individuals	Markers	QTL	Epistasis	Heritability
Base	600	250	55	0.35	0.35
Increment	400(0.74)	150(−0.16)	45(−0.12)	0.25	0.15(0.78)
*Δ*	296	−24	−5.4	0.25	0.12
Base+Δ	896	226	50	0.6	0.47
Base+2Δ	1192	202	44	0.85	0.59
Base+3Δ	1488	178	39	1	0.71
Base+4Δ	1784	154	33	1	0.83
Base+5Δ	2080	130	28	1	0.95
Base+6Δ	2376	106	23	1	1
Base+7Δ	2672	82	17	1	1
Base+8Δ	2968	58	12	1	1
Base+9Δ	3264	34	6	1	1

**Table 8:** Coordinates of the steepest ascent for the number of individuals, number of markers, number of QTL, proportion of epistasis, and the degree of heritability for the additional runs when the response is the mean accuracy difference between the SVM and BLUP, and the corresponding mean accuracy for BLUP, SVM, and for SVM–BLUP

Factor Level	Individuals	Markers	QTL	Epistasis	Heritability	$r_{BLUP}$	$r_{SVM}$	$r_{SVM}-r_{BLUP}$
1	896	226	50	0.60	0.47	0.37	0.38	0.01
2	1192	202	44	0.85	0.59	0.26	0.29	0.03
3	1488	178	39	1	0.71	0.00	0.23	0.23
4	1784	154	33	1	0.83	0.01	0.31	0.30
5	2080	130	28	1	0.95	0.01	0.46	0.45
6	2376	106	23	1	1	0.01	0.55	0.54
7	2672	82	17	1	1	0.00	0.62	0.62
8	2968	58	12	1	1	0.01	0.73	0.72
9	3264	34	6	1	1	0.00	0.98	0.98

After adjusting for the operability regions of the five factors, the second set of experimental conditions for $\left[r_{SVM}-r_{BLUP}\right]$ and associated responses indicated that the increased number of individuals, proportion of genetics due to epistasis, and h, and the decreased number of markers and QTL produced improved responses. As the number of segregating progeny, the proportion of epistasis, and h increase, the advantage of using the SVM method instead of BLUP increases. The total number of factor combinations evaluated to find the maximum $\left[r_{SVM}-r_{BLUP}\right]$ was <25, instead of the 405 which would be required to describe the entire surface (*Response surfaces*).

The estimated regression coefficients for the averaged response $y_{2}=r_{BLUP}$ are listed in the estimated regression line:

$$\hat{y_{2}}=0.462+0.039ind-0.005m+0.025QTL-0.285epi+0.144h,$$ (27)

indicating that increasing the number of progeny, number of QTL, and h, and decreasing the number of markers and the proportion of genetic variance due to epistasis should increase the response. The estimated coefficients are the smallest for the number of markers and QTL, so these factors are not as influential as the other factors. Because the largest estimated coefficient in absolute value is associated with epistasis, the basis is epistasis and the corresponding step size is =−0.25 for the response of accuracy of prediction using BLUP. Note that the step size of the basis has a negative value here because the estimated regression coefficient for epistasis is negative. The step size for epistasis =0.25 in natural units corresponds to 0.25/0.15=1.6667 in coded units. For the other four factors, the step sizes in coded units are

$$\left(\frac{0.039}{-0.285}\right)\left(-1.6667\right)=0.228$$ (28)

for individuals,

$$\left(\frac{-0.005}{-0.285}\right)\left(-1.6667\right)=-0.029$$ (29)

for markers,

$$\left(\frac{0.025}{-0.285}\right)\left(-1.6667\right)=0.146$$ (30)

for QTL, and

$$\left(\frac{0.144}{-0.285}\right)\left(-1.6667\right)=0.842$$ (31)

for heritability.

Factor levels for the next set of analyses resulted in decreasing factor levels for epistasis and number of markers (Table 9 and Table 10) for maximizing $r_{BLUP}.$ The optimum response for averaged $r_{BLUP}$ occurs when the genetic variance is explained by purely the additive effects. For both responses ($y_{1}$ and $y_{2}$), the response increases when the number of segregating progeny and heritability increase. For both responses, it is beneficial to have markers which explain the phenotypic variation, and it does not improve prediction accuracies when markers which are not associated with the phenotypic variability are added. The total number of factor combinations needed to find the maximum responses for $r_{BLUP}$ was 21, ∼5% of what would be required to describe the entire response surface.

**Table 9:** Base, step size in natural units, and the coordinates of the steepest ascent for the number of individuals, number of markers, number of QTL, proportion of epistasis, and the degree of heritability for response $r_{BLUP}$

	Individuals	Markers	QTL	Epistasis	Heritability
Base	600	250	55	0.35	0.35
Increment	400(0.228)	150(−0.029)	45(0.146)	−0.25	0.15(0.842)
*Δ*	91	−4.4	6.6	−0.25	0.13
Base+Δ	691	246	62	0.10	0.48
Base+2Δ	782	241	68	0	0.61
Base+3Δ	873	237	75	0	0.74
Base+4Δ	964	232	81	0	0.87
Base+5Δ	1055	228	88	0	1

**Table 10:** Coordinates of the steepest ascent for the number of individuals, number of markers, number of QTL, proportion of epistasis, and the degree of heritability for the additional runs when the response is the mean accuracy for BLUP, and the corresponding mean accuracy for BLUP

	Individuals	Markers	QTL	Epistasis	Heritability	BLUP
Run 1	691	246	62	0.1	0.48	0.62
Run 2	782	241	68	0	0.61	0.74
Run 3	873	237	75	0	0.74	0.83
Run 4	964	232	81	0	0.87	0.92
Run 5	1055	228	88	0	1	1

## Discussion

### Some considerations about the factors

In 2014, we conjectured that differences between estimated prediction accuracies of linear-model and algorithmic methods could be used as a computational diagnostic for an epistatic genetic architecture. Huang *et al.* (2012) had previously alluded to failure of linear model-based methods as an indicator of epistatic genetic architectures. Thus, we had an obligation to investigate the hypothesis using a more thorough and systematic approach. We learned that the response surface for the computational diagnostic, ($r_{SVM}-r_{BLUP}$), is flat except in the vicinity of maximum values for the proportional contribution of epistasis to genetic variance, and the proportional contribution of genetic variance to phenotypic variability. To our knowledge there are no known quantitative traits that exhibit such genetic architectures.

Before designing the experiments for the steepest ascent/descent procedure, it is important to evaluate some initial experiments where we determine which factors might be important and which can be excluded from the model. We also have to define the region in which the factors can affect the response, also known as the operability region.

The initial choices of the factors we consider and their range of values can have a large impact on the speed of approaching the optimal response, and whether the optimum is reached. For example, for the factor temperature we can choose the range to be between 1 °F and 100 °F, or we can convert to the Celsius scale which will lead to a range between −17 °C and 38 °C. The estimated regression coefficients will be different depending on the temperature scale. Using different ranges of factors only influences the magnitude of the regression coefficients, not the sign of the regression coefficients. This implies that using a different range for the factors would not modify the direction in which we are moving along the path, but it would change the speed of the movement relative to the scale used.

Another design aspect to consider is the choice of the metric for the response. Especially when the range of the response is large, it is useful to transform the response. One of the most commonly used transformations is the Box–Cox power transformation (Box and Cox 1964). The transformed response, *w* is defined as

$$w=\frac{y^{\lambda }-1}{\lambda },$$ (32)

where *y* is the untransformed response and *λ* is the power parameter. For example, λ=−1 results in a reciprocal transformation, λ=0.5 results in a square root transformation, and since ${\text{lim}}_{\lambda \to 0}\left[\left(y^{\lambda }-1\right)/\lambda \right]=\text{ln}y,$ λ=0 results in a logarithmic transformation. The power parameter *λ* can be estimated via maximum likelihood. This rank-preserving transformation is also useful when we need to stabilize the variance of the response, or when the residual variance does not satisfy the normal assumptions. It is possible that the response surface has multiple peaks, and then more than one combination of the design variables satisfies the condition for having the optimal response. It is also likely that the range of starting values do not include the global peaks, but in the procedure of steepest descent (or ascent) we would arrive in the required range.

### Application of RSMs for evaluation of GP methods

Originally, RSMs (Box and Wilson 1951) were developed to find combinations of controlled conditions to maximize output of industrial processes (Naylor 1969). Myers *et al.* (1989) summarized the extensive applications of RSMs for systems engineering; Bezerra *et al.* (2008) summarized applications in analytical chemistry; and RSMs have been applied to systems of interest to biologists, including pharmaceutical production (Koyamada *et al.* 2004) and fermentation (Zhang *et al.* 2010). Our own experiences in consulting have revealed a misperception that RSMs can only be applied to systems where the factors and factor levels can be explicitly planned and controlled as fixed-effect treatments. However, RSMs have been shown to be efficient for determining optimal conditions of complex systems where many of the factors are represented as random samples or unknown factors, *e.g.*, ecosystem impacts on growth and development of individuals (Menke 1973). Herein, we demonstrated that the steepest ascent RSM also provides an efficient approach to identify conditions that affect prediction accuracies of a machine learning and a BLUP method. The purpose here is not to conduct many different experiments, but to evaluate the sensitivity of the GP models to the underlying genetic architecture and design factors with a limited number of experiments. To our knowledge, this is the first report of RSM for GP methods and a demonstration of how to find factor combinations that are responsible for maximizing prediction accuracies using an efficient number of experimental analyses. Further, we demonstrated that the steepest ascent RSM also provided useful information about the response surface without the burden of evaluating every possible combination of factors and factor levels.

Even though we demonstrated RSM using GP methods, the methodology can be applied during development of any novel data analyses or computational methods, although this proposition requires more research. There are some aspects of our application of an RSM that need to be emphasized. First, steepest ascent represents only one of many RSMs, and use of a fractional factorial represents only one of many efficient experimental designs. We chose these because our goal was to find a set of factor levels in which the responses were maximized and we had some prior information about possible factors. The set of factors that we investigate might not be the only set associated with maximizing responses. For example, in case we find two sets of factor levels that are associated with the maximum response, suggesting that there might be a ridge of maximum responses, then a future ridge analysis RSM could be justified. Also, if we had some prior knowledge that our maximum responses were likely to occur at intermediate levels for our sets of factors, the central composite design would provide a more efficient design than the half-fractional factorial for the initial set.

Before designing and conducting an initial experiment, knowledge about factors and factor levels should be incorporated into defining the operability regions, *i.e.*, ranges of values of the factors that are attainable. From prior publications, we were aware that genetic architecture of the trait (number of QTL, epistasis among QTL, and interactions of QTL with environments), population structure, relationships between training and validation data sets, number of genetic markers, heritability, and number of individuals (lines, varieties, and hybrids) could affect the predictive ability of GP methods. We were primarily interested in the potential of the computational diagnostic ($r_{SVM}-r_{BLUP}$) for detection of epistatic genetic architectures involving a large, but finite, number of QTL (Clowers *et al.* 2010; Howard *et al.* 2014). Further investigation is needed on larger numbers of QTL especially for traits like yield or biomass where the infinitesimal model is more appropriate. Also, since it is likely that the diagnostic will detect interactions of QTL with environments, we decided to avoid this confounded interpretation for this investigation. The operability region for epistasis was restricted to two-way interactions. Higher order interactions were not considered because Cooper *et al.* (2002) pointed out that response to selection on an adaptive surface is unlikely to happen if the average number of interactions among QTL is much more than two. Crops have been responding to selection for at least 100 yr. Since it is trivial for simulation to produce any level of epistasis, the two-way interactions were simulated to represent the full range of contributions to genetic variance. Likewise, the operability region for heritability was simulated to represent the full range of contributions to phenotypic variance. We also kept the population structure and relationships among training and validation subsets consistent and simple to avoid confounded interpretations of the proposed diagnostic.

With the emergence of genotyping-by-sequencing technologies, the operability region for the number of markers could have been much larger; however, given maximal linkage disequilibrium (LD) in the population structure, the operability region need only include sufficient numbers to assure some redundancy among genotypic information. Indeed, we found that if the LD between markers and QTL is complete, then prediction accuracies are not improved with any additional markers. This outcome will likely not change with more complex population structures for finite numbers of segregating QTL.

The operability region for number of individuals was based on a possible number of DH progeny with application of DH technologies to *F*_2_ progeny from a single cross of inbred lines in a crop such as maize. While 2000 DH progeny are possible, producing such numbers is unlikely without significant resource allocations. Thus, from a practical perspective, it is highly unlikely that the diagnostic can be employed routinely because the maximum values for the diagnostic occurred with large numbers of progeny in which the genetic architecture is comprised entirely of epistatic variance and broad sense heritability is unity. Also, the large difference in accuracy between SVM and BLUP occurs at the extreme of pure epistatic variance, which is a genetic architecture that has not been described for any trait. Thus, the reader should be aware that the GP models can have a similar accuracy in cases when the genetic architecture consists of a high proportion of epistatic variance, and only comparing prediction accuracies might not be a useful method to infer genetic architecture.

Lastly, the reader will note that prediction accuracy results are not exactly the same as results previously reported by Howard *et al.* (2014). In the prior report, we simulated *F*_2_ and backcross populations where the linkage groups had different lengths. This report is based on DH lines with linkage groups that consist of the same numbers of marker loci per linkage group and the same recombination among adjacent marker loci within linkage groups. Consequently, the LD among the simulated QTL was not the same in the two studies. The inconsistencies between the two reports are small, but the results suggest that LD among QTL, population structures, and genomic architecture also will influence prediction accuracies. In particular, it is likely that because of LD among QTL in our first set of simulations we unintentionally produced a second source of nonlinear interactions, *i.e.*, pseudooverdominance.

## Supplementary Material

Supplemental material is available online at www.g3journal.org/lookup/suppl/doi:10.1534/g3.117.044453/-/DC1.
